# Osthol Ameliorates Kidney Damage and Metabolic Syndrome Induced by a High-Fat/High-Sugar Diet

**DOI:** 10.3390/ijms22052431

**Published:** 2021-02-28

**Authors:** Fernando E. García-Arroyo, Guillermo Gonzaga-Sánchez, Edilia Tapia, Itzel Muñoz-Jiménez, Lino Manterola-Romero, Horacio Osorio-Alonso, Abraham S. Arellano-Buendía, José Pedraza-Chaverri, Carlos A. Roncal-Jiménez, Miguel A. Lanaspa, Richard J. Johnson, Laura Gabriela Sánchez-Lozada

**Affiliations:** 1 Department of Cardio-Renal Physiopathology, INC Ignacio Chávez, Mexico City 14080, Mexico; ggonzaga49@gmail.com (G.G.-S.); edilia.tapia@cardiologia.org.mx (E.T.); itzelmj0707@gmail.com (I.M.-J.); linomr9@gmail.com (L.M.-R.); horacio.osorio@cardiologia.org.mx (H.O.-A.); abraham.arellano@cardiologia.org.mx (A.S.A.-B.); 2 Department of Biology, Faculty of Chemistry, National Autonomous University of Mexico (UNAM), Mexico City 04510, Mexico; pedraza@unam.mx; 3 Division of Renal Diseases and Hypertension, University of Colorado, Aurora, CO 80045, USA; CARLOS.RONCAL@cuanschutz.edu (C.A.R.-J.); Miguel.LanaspaGarcia@cuanschutz.edu (M.A.L.); Richard.Johnson@cuanschutz.edu (R.J.J.)

**Keywords:** metabolic syndrome, renal lipotoxicity, fructokinase, coumarins

## Abstract

Excessive intake of fructose results in metabolic syndrome (MS) and kidney damage, partly mediated by its metabolism by fructokinase-C or ketohexokinase-C (KHK-C). Osthol has antioxidant properties, is capable of regulating adipogenesis, and inhibits KHK-C activity. Here, we examined the potential protective role of osthol in the development of kidney disease induced by a Western (high-fat/high-sugar) diet. Control rats fed with a high-fat/high-sugar diet were compared with two groups that also received two different doses of osthol (30 mg/kg/d or 40 mg/kg/d body weight BW). A fourth group served as a normal control and received regular chow. At the end of the follow-up, kidney function, metabolic markers, oxidative stress, and lipogenic enzymes were evaluated. The Western diet induced MS (hypertension, hyperglycemia, hypertriglyceridemia, obesity, hyperuricemia), a fall in the glomerular filtration rate, renal tubular damage, and increased oxidative stress in the kidney cortex, with increased expression of lipogenic enzymes and increased kidney KHK expression. Osthol treatment prevented the development of MS and ameliorated kidney damage by inhibiting KHK activity, preventing oxidative stress via nuclear factor erythroid 2-related factor (Nrf2) activation, and reducing renal lipotoxicity. These data suggest that the nutraceutical osthol might be an ancillary therapy to slow the progression of MS and kidney damage induced by a Western diet.

## 1. Introduction

The role of diet in metabolic and kidney diseases is increasingly recognized, especially the Western-type diet, in which intake of refined and processed foods, red meats, fat, and sugary beverages is high, while consumption of fibers, fruits, and vegetables is low [1,2]. In addition to the increased risk for metabolic syndrome (MS) [2,3], a Western diet, and especially its sugar component, also increases the risk for non-alcoholic fatty liver disease (NAFLD) and type 2 diabetes (D2M) [4,5,6,7,8,9].

Chronic kidney disease (CKD) is also linked with metabolic disorders, including dyslipidemia, obesity, hypertension, hyperglycemia, and cardiovascular diseases [10]. Indeed, metabolic syndrome is one of the most important risk factors for CKD [11]. Other studies have reported that the severity of metabolic syndrome (MS) is strongly linked with cardiovascular diseases and CKD and that changes in lifestyle could prevent renal involvement [12,13]. Potential mechanisms that might link MS and CKD include endothelial dysfunction, insulin resistance, inflammation, mitochondrial dysfunction, hyperuricemia, and oxidative stress [14]. Additional kidney-specific mechanisms may also include increased renal Na^+^ reabsorption, intrarenal fat accumulation, renin-angiotensin-aldosterone system (RAAS) activation, sympathetic nervous system (SNS) activation, and lipotoxicity [15].

A Western diet also contains a high content of refined sugars, of which fructose is a significant component. Excessive intake of fuctose has been shown to cause hyperuricemia [16], MS, cardiovascular damage, hypertension, a decrease in the glomerular filtration rate (GFR), and renal disease [7,17,18,19]. Fructose induces its metabolic effects primarily from the nucleotide degradation that results following metabolism of fructose by the C isoform of KHK. This enzyme lacks a negative feedback mechanism, thereby phosphorylating fructose rapidly, depleting cellular phosphate and ATP and activating the purine degradation pathway to induce uric acid production [7,18,20]. In this regard, mice lacking KHK have been shown to be protected from developing metabolic syndrome by diets containing high concentrations of fructose [21].

Recently, a study evaluated a variety of natural compounds for their ability to inhibit KHK activity. One promising compound identified was osthol, which is a very concentrated coumarin isolated from the plant *Angelica archangelica* [22]. Osthol showed KHK-C inhibitory activity and blocked fructose-induced ATP depletion and the concomitant increase in uric acid synthesis in HepG2 cells [22]. Osthol was also shown to protect mice with hereditary fructose intolerance (HFI) due to the genetically induced absence of aldolase B through its ability to block KHK [23]. Osthol has also been reported to block kidney fibrosis, ischemia-reperfusion injury [24,25], and acute kidney injury, likely by inhibiting inflammation resulting from activation of nuclear factor κ-β and Janus kinase/signal transducers and activators of transcription (JAK2/STAT3) pathways [26,27,28,29,30]. Osthol provides antioxidant protection by promoting expression of nuclear translocation of nuclear factor erythroid 2-related factor (Nrf2) [28].

Therefore, the objective of this work was to determine whether osthol can prevent kidney disease and metabolic syndrome induced by a high-fat/high-sugar diet.

## 2. Results

### 2.1. Body Weight, Food Intake, Beverage Intake and Osthol Dosing

**Figure 1:**
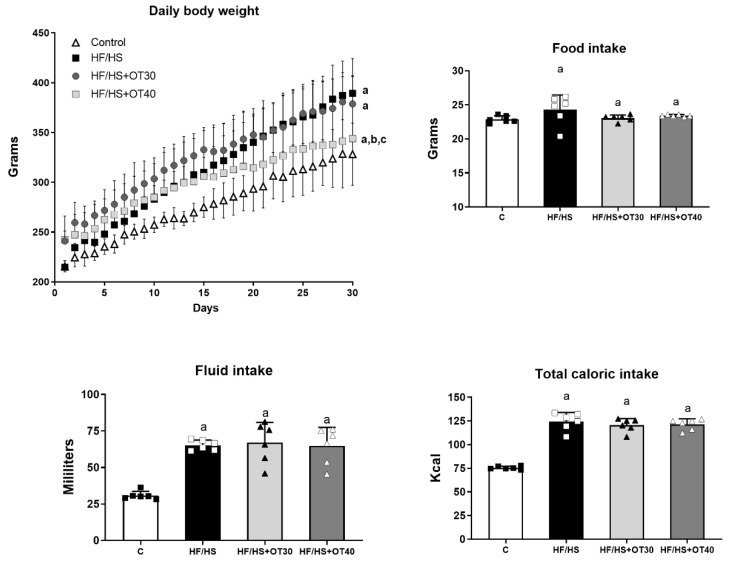
Effects of high-fat/high-sugar (HF/HS) diet and osthol administration on body weight, total intake of food, fluids, and calories. Results are presented as te mean ± SD and analyzed by one-way ANOVA. Post hoc analysis was performed using Bonferroni’s test. a: *p* < 0.05 vs. control.

The experimental strategy of food control was effective, since we did not observe significative changes in the mean food consumption among groups (Figure 1). Fluid intake increased similarly in those groups receiving a sweetened beverage (SB) in comparison to the control group receiving tap water (Figure 1). All groups receiving a high-fat/high-sugar (HF/HS) diet had a significantly higher total caloric intake compared to groups on a regular diet (Figure 1). We observed that HF/HS and OT30 groups showed higher and significative body weight increments vs. C and OT40 groups after 4 weeks of follow-up (Figure 1). The doses of osthol that rats received in OT30 and OT40 groups were calculated from the daily food intake, and the mean doses were 23.03 ± 0.18 mg/day and 28.76 ± 0.12 mg/day, respectively, and were statistically different.

### 2.2. Renal Effects of an HF/HS Diet

**Figure 2:**
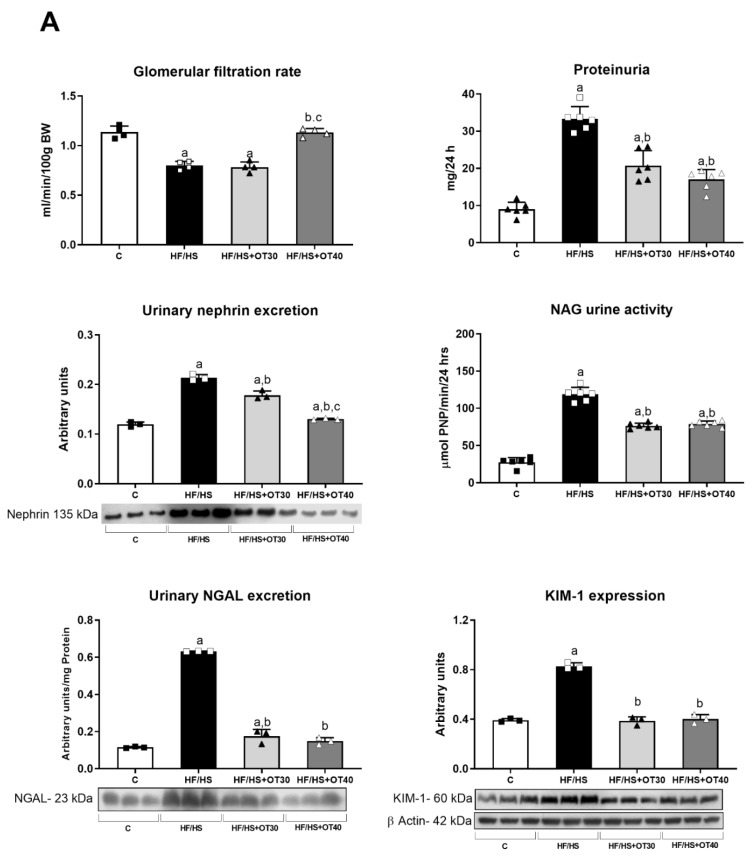

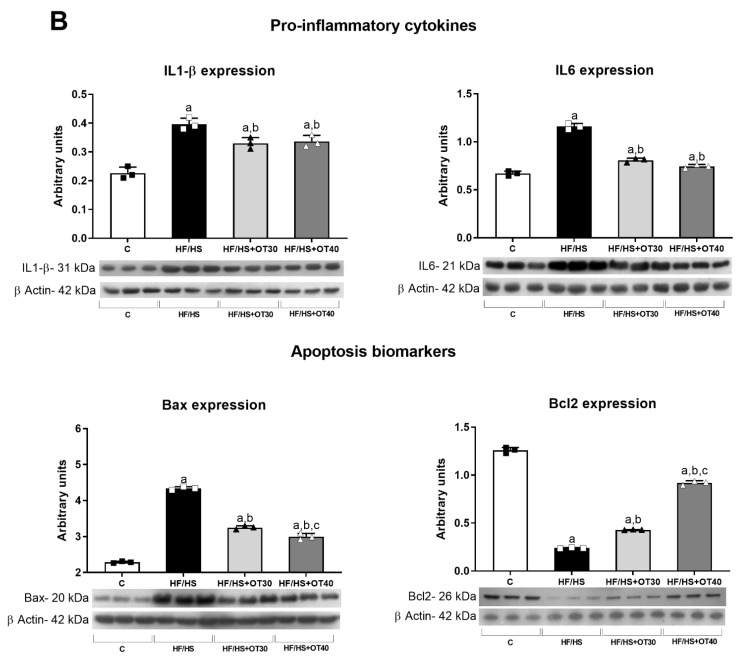
Effects of HF/HS diet and osthol administration on renal function and markers of renal damage. (**A**) Glomerular filtration rate (GFR), proteinuria, urinary nephrin excretion, N-acetyl-β-D-glucosaminidase (NAG) urine activity, urinary neutrophil gelatinase associated-lipocalin (NGAL) urinary excretion, and kidney injury molecule (KIM)-1 renal cortex expression. (**B**) Pro-inflammatory cytokine expression in the renal cortex (IL-1β and IL-6) and apoptosis biomarker expression (BAX and Bcl2). For Western blotting, 3 randomly selected samples per group were analyzed. Results are presented as the mean ± SD and analyzed by one-way ANOVA. Post hoc analysis was performed using Bonferroni’s test. a: *p* < 0.05 vs. Control; b: *p* < 0.05 vs. HF/HS; c: *p* < 0.05 vs. HF/HS + OT30).

After the end of the follow-up, the GFR was measured transdermally with 5/6-fluorescein isothiocyanate (FITC)-sinistrin. We observed a significant decrease in renal function in HF/HS and OT30 groups. The highest dose of osthol used, in the OT40 group, prevented a fall in the GFR (Figure 2A). Rats treated with an HF/HS diet also developed proteinuria, which was prevented by both doses of osthol. Likewise, osthol administration (OT30 and OT40) partially prevented tubular damage measured by N-acetyl-β-D-glucosaminidase (NAG) and neutrophil gelatinase associated-lipocalin (NGAL) urine excretion, as well as kidney injury molecule (KIM)-1 expression in comparison to C and HF/HS groups (Figure 2B). Additionally, pro-inflammatory cytokine expression (IL-1β and IL-6) and apoptosis biomarkers (BAX and Bcl2) were overexpressed in the renal cortex of HF/HS rats, and this was partially prevented by both doses of osthol (Figure 2B).

### 2.3. Systemic Markers of Metabolic Alterations and Blood Pressure

**Figure 3:**
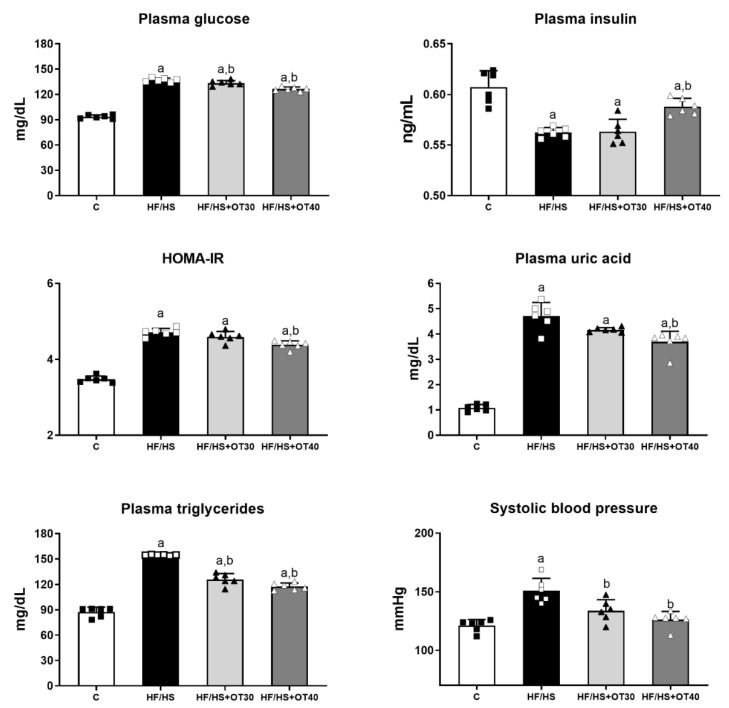
Systemic and renal effects of HF/HS diet. Plasma levels of glucose, insulin, uric acid, triglycerides, blood pressure, and homeostasis model assessment–immunoreactivity (HOMA-IR) values. Results are presented as the mean ± SD and analyzed by one-way ANOVA. Post hoc analysis was performed using Bonferroni’s test. a: *p* < 0.05 vs. C; b: *p* < 0.05 vs. HF/HS.

The HF/HS group showed a significant increase in plasma glucose, insulin, uric acid, and triglycerides compared to the control group. Osthol administration partially and dose-dependently decreased plasma concentrations of glucose and triglycerides. Osthol also decreased plasma uric acid and plasma insulin, but only the OT40 group reached statistical significance compared to the HF/HS group (Figure 3). The homeostasis model assessment–immunoreactivity (HOMA-IR) was calculated from fasting plasma glucose and insulin levels using the validated equation HOMA IR = serum insulin (mmol/L) × (blood glucose (mmol/L)/22.5. We observed that HOMA-IR increased in the HF/HS group: osthol at the highest dose (HF/HS + OT40) partially prevented this effect. Skeletal muscle sensitivity to insulin was also improved by osthol as glucose transporterGLUT4, as well as the insulin receptor substrates 1 and 2 (IRS1, and IRS2) expression increased by the highest dose (Supplementary Materials, Figure S1). Systolic blood pressure significantly increased after 4 weeks of HF/HS diet intake, and both doses of osthol prevented the rise in blood pressure.

### 2.4. Fructokinase Expression and Activity Assessment and Markers of Fructokinase Activation

**Figure 4:**
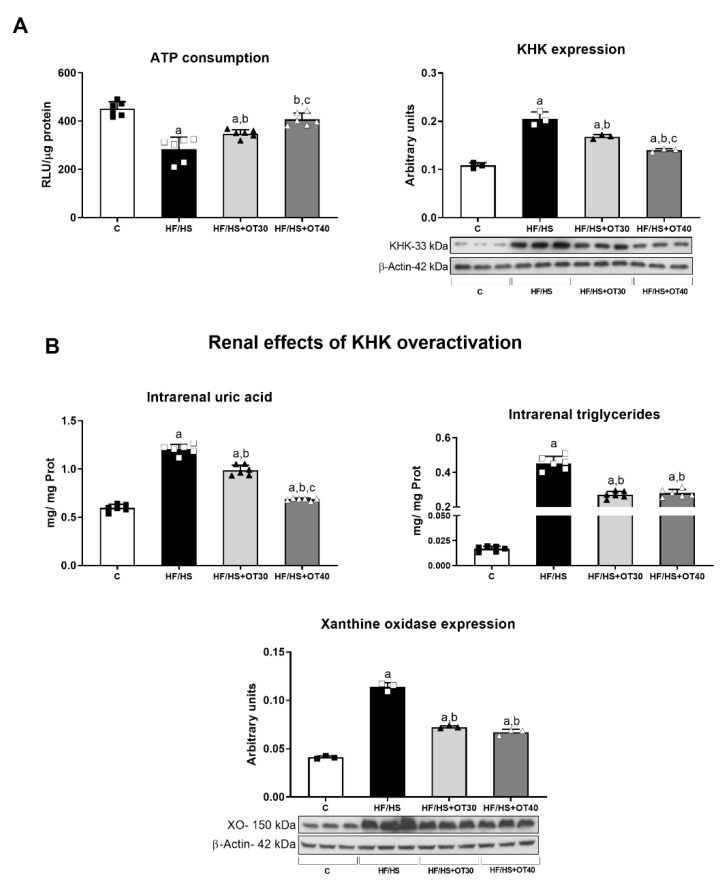
Assessment of the fructokinase (KHK) axis. (**A**) Activity and expression of KHK activity in the kidney and (**B**) intrarenal levels of uric acid and triglycerides and renal xanthine oxidase (XO) expression. For Western blotting, 3 randomly selected samples per group were analyzed. Results are presented as the mean ± SD and analyzed by one-way ANOVA. Post hoc analysis was performed using Bonferroni’s test. a: *p* < 0.05 vs. C; b: *p* < 0.05 vs. HF/HS; c: *p* < 0.05 vs. HF/HS + OT30.

We measured KHK activity indirectly using ATP consumption as a proxy, as previously described. We observed that ATP consumption was higher in renal cortex homogenates obtained from the HF/HS group in comparison to the C group. Osthol dose-dependently and partially reduced ATP consumption, indicating a lower activity of KHK in those groups. We also measured ATP concentrations in renal cortex homogenates and confirmed that the HF/HS group had reduced tissue ATP concentrations. Osthol treatment dose-dependently prevented the decrease in ATP concentrations in the renal cortex. Consistent with these findings, an HF/HS diet significantly increased renal cortex KHK expression and osthol dose-dependently prevented KHK overexpression (Figure 4). We also measured intrarenal uric acid and triglycerides, both parameters associated with KHK activation. The HF/HS group showed a significant increase in both intrarenal uric acid and triglycerides. Osthol administration dose-dependently ameliorated the increase in uric acid, and both doses reduced intrarenal TGs similarly (Figure 4). Renal xanthine oxidase (the limiting enzyme in uric acid synthesis) was overexpressed in the HF/HS group, and this was prevented in rats receiving both doses of osthol (OT30 and OT40) (Figure 4).

### 2.5. Assessment of Renal Lipid Accumulation

**Figure 5:**
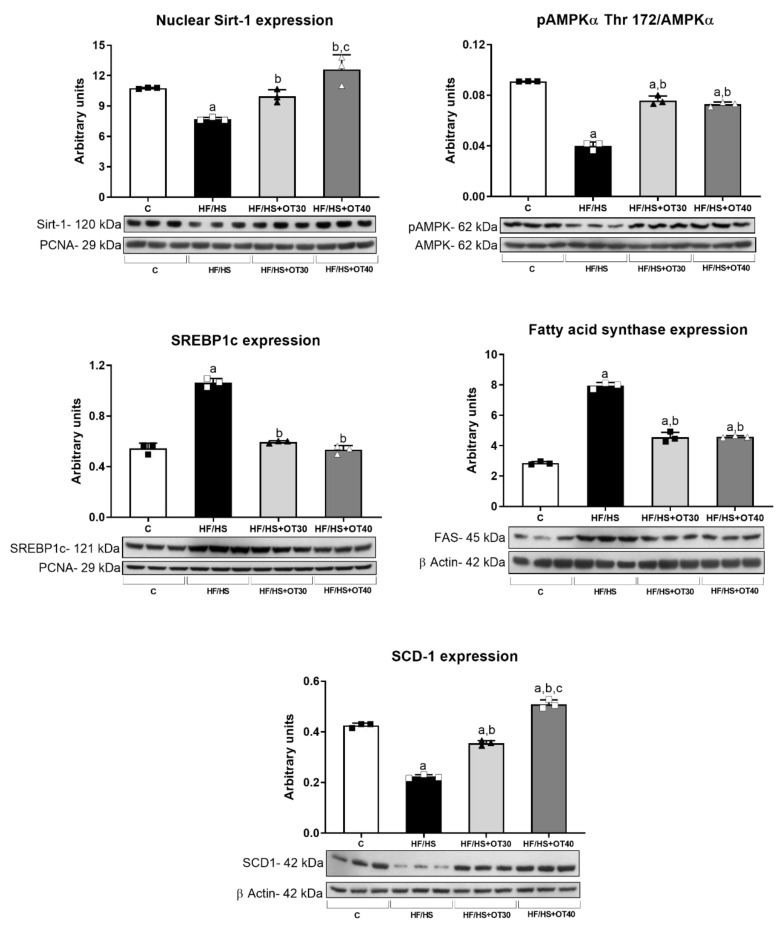
Lipogenesis studies. Renal expression of Sirt-1, stearoyl CoA desaturase 1 (SCD-1), AMPKα, phospho-AMPKα Thr 172, SREBP1c, and fatty acid synthase (FAS). For Western blotting, 3 randomly selected samples per group were analyzed. Results are presented as the mean ± SD and analyzed by one-way ANOVA. Post hoc analysis was performed using Bonferroni’s test. a: *p* < 0.05 vs. C; b: *p* < 0.05 vs. HF/HS; c: *p* < 0.05 vs. HF/HS + OT30.

Nuclear Sirt-1, AMPKα and phospho-AMPKα Thr 172, nuclear SREBP1c, and fatty acid synthase (FAS) expression were evaluated in renal cortex homogenates. Sirt-1 and stearoyl CoA desaturase 1 (SCD-1) showed lower expression in the HF/HS group compared to the C group, and osthol dose-dependently prevented this fall (Figure 5). AMPKα expression was not different among groups; however phospho-AMPKα Thr 172 expression was lower in the HF/HS group, and both doses of osthol maintained phospho AMPKα Thr 172 expression (Figure 5). In addition, SREBP1c (adipogenic transcription factor) and fatty acid synthase (FAS) expression increased in the HF/HS group. Both doses of osthol similarly prevented the overexpression of these proteins (Figure 5).

### 2.6. Oxidative Stress

**Figure 6:**
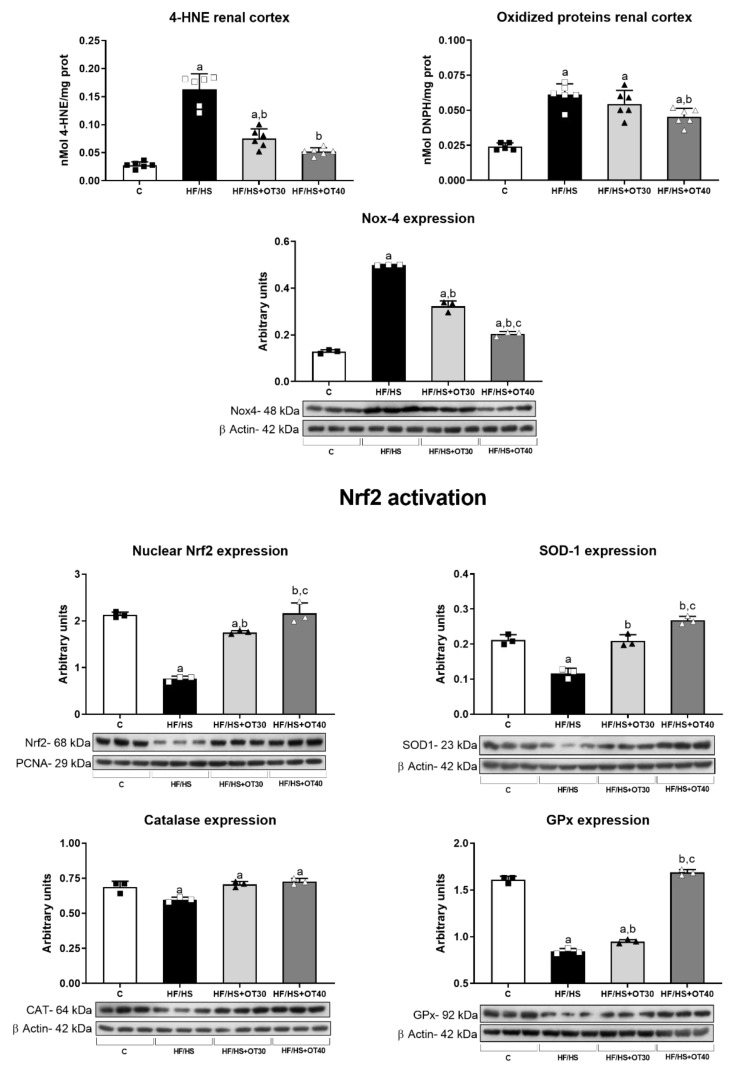
Oxidative stress. Shown are measurements of lipid peroxidation (4-hydroxynonenal (4-HNE)), protein carbonylation, and levels of expression of Nox-4, nuclear factor erythroid 2-related factor (Nrf2), and antioxidant enzymes (SOD-1, CAT, and GPx) in the renal cortex. For Western blotting, 3 randomly selected samples per group were analyzed. Results are presented as the mean ± SD and analyzed by one-way ANOVA. Post hoc analysis was performed using Bonferroni’s test. a: *p* < 0.05 vs. C; b: *p* < 0.05 vs. HF/HS; c: *p* < 0.05 vs. HF/HS + OT30.

The quantification of protein carbonylation (marker of protein oxidation) and 4-hydroxynonenal (4-HNE; a marker of lipid peroxidation) indicated that renal oxidative stress significantly increased in the HF/HS group vs. the C group. Both doses of osthol prevented the increase in 4-HNE. Nevertheless, only OT40 prevented the increase in protein carbonylation compared to the C, HF/HS, and OT30 groups (Figure 6). Nox-4 is a source of oxidative stress in renal tissue. We found that Nox-4 was overexpressed in HF/HS. Osthol administration dose-dependently prevented Nox-4 overexpression (Figure 5). Additionally, we assessed renal expression of Nrf2 by Western blotting. The HF/HS group showed lower expression in comparison to control rats. Osthol dose-dependently maintained the nuclear expression of Nrf2 transcription factor, and the higher dose normalized the expression to levels observed in control rats (Figure 6). As Nrf2 nuclear translocation is involved in the activation of the antioxidant response, we evaluated the expression ofsuperoxide dismutase-1(SOD-1), catalase (CAT), and glutathione peroxidase (GPx) in renal cortex nuclear extracts. In concordance with a lower Nrf2 nuclear translocation induced by an HF/HS diet, the expression of SOD-1, CAT, and GPx also significantly reduced. Likewise, osthol dose-dependently prevented the decrement in the expression of SOD-1 and GPx, while both doses successfully maintained CAT expression (Figure 6).

## 3. Discussion

Here, we demonstrated that a Western diet induces metabolic syndrome and kidney injury, the latter characterized by oxidative stress and reduced renal function. We also demonstrated evidence of the activation of fructokinase (KHK), as noted by increased renal expression, increased activity, and increased end products (intracellular uric acid, reduced intrarenal ATP levels). Remarkably, the administration of osthol, a nutraceutical that has been shown to block KHK activity [22], resulted in the amelioration of metabolic syndrome and the prevention of kidney injury associated with inhibition of KHK expression and activity. Thus, these results suggest that osthol might be useful in the treatment of metabolic and renal disorders induced by diets high in sugar and fat.

An important feature of metabolic syndrome is the development of overweight/obesity. Not surprisingly, a HF/HS Western diet induced weight gain. Osthol administration partially prevented (~8%) weight gain despite rats having similar caloric intake. These findings are in agreement with previous studies [31,32,33,34,35,36,37,38,39,40,41,42,43,44,45] and suggest that a lower increase in body weight is not due to an anorexigenic effect and likely is more related to the ability of osthol to modulate lipogenesis by activating the peroxisome proliferator-activated receptors (PPARs) α, β, and γ [46], as well as by inhibiting KHK-C [23]. We also confirmed that osthol could partially prevent the rise in fasting glucose, insulin, triglycerides, uric acid, and insulin resistance. These latter effects may be related to the prevention of KHK overexpression and overactivity in the liver (Supplementary Materials, Figure S2), as the liver KHK expression appears to play a key role in driving all of the features of metabolic syndrome associated with sugar [47]. At the kidney level, the HF/HS diet induced a 20% fall in the GFR that was associated with proteinuria and increased expression of KIM-1 in the kidney cortex as well as increased urinary excretion of nephrin, NAG, and NGAL, suggesting glomerular and tubular damage. Osthol treatment, especially the highest dose, provided significant benefit.

The accumulation of lipid intermediary or final products in tissues and organs other than adipose tissue is called lipotoxicity. Diets high in fats and sugar promote renal lipotoxicity associated with renal inflammation and apoptosis [48,49]. In agreement with such studies, we found that the HF/HS diet induced overexpression of apoptosis biomarkers and pro-inflammatory cytokines. The proximal tubule epithelium is a high-energy-demanding tissue; therefore, it critically depends upon fatty acid oxidation to achieve its energy demands as this metabolic route provides more molecules of ATP per carbon atom in comparison to glucose oxidation. Excess of systemic free fatty acids (FFA) leads to renal proximal tubule overload exceeding the beta-oxidative capacity and resulting in the accumulation of intracellular triglycerides and toxic lipid metabolites [50]. In the present study, we observed that rats fed the HF/HS diet had significant accumulation of triglycerides in the renal cortex, suggesting lipotoxicity, and both doses of osthol partially prevented this. In addition, osthol also partially prevented the overexpression of pro-inflammatory cytokines and partially rescued the expression of apoptosis biomarkers.

In addition to FFA overload, altered renal lipid metabolism can also cause intrarenal fat accumulation. In effect, we found that the HF/HS diet increased lipogenesis, as suggested by the significantly higher nuclear expression of the adipogenic transcription factor SREBP-1 and its target, fatty acid synthase (FAS). On the other hand, Sirt1 and phospho-AMPKα Thr 172 expression, which are both associated with fatty acid beta-oxidation, were significantly reduced. Moreover, rats fed the HF/HS diet demonstrated a significant decrease in the expression of stearoyl CoA desaturase 1 (SCD1) in the renal cortex, as has been previously described in mice receiving a high-fat diet [51]. SCD1 is the enzyme that produces monounsaturated fatty acids (MUFAs) from saturated fatty acids (SFAs). MUFAs are less toxic than SFAs as the latter promote endoplasmic reticulum stress and apoptosis in proximal tubule cells [51]. Osthol treatment provided therapeutic benefit by dose-dependently preventing such lipid metabolic disturbances.

Increased fructose metabolism mediated by KHK in renal tubules is associated with glomerular alterations, proteinuria, hypertension, and oxidative stress [20,39,52,53]. The HF/HS diet significantly increased KHK expression and activity in the renal cortex, along with a significant reduction in ATP concentration, and increased tissue uric acid. These alterations were associated with a reduced GFR, proteinuria, and elevated urinary NAG, NGAL, and KIM-1. Osthol treatment dose-dependently prevented KHK overexpression and overactivity. Such effects were in parallel with the preservation of renal tissue ATP, prevention of the increment in tissue uric acid as well as with the maintenance of renal function, and an almost complete normalization of the renal lesion markers. In addition, fructose metabolism by KHK also stimulates adipogenesis and reduces fatty acid beta-oxidation [54], therefore aggravating renal lipotoxicity.

Oxidative stress plays a primary role in the development of metabolic syndrome and kidney damage [55,56]. We found that a Western diet induced lipid and protein oxidation in the renal cortex. This oxidative damage may be mediated by overactivation of Nox4, which was inversely related to a lower expression of renal antioxidant enzymes (SOD-1, CAT, and GPx). Others have found that diets high in sugar also induce the overactivation of Nox and xanthine oxidase in the hypothalamus, concurrent with higher oxidative stress, suggesting that the activation of pro-oxidant enzymes shifts redox homeostasis toward oxidative reactions. Osthol has been reported to promote the expression of the transcription factor Nrf2, inducing increased expression of antioxidant enzymes [56,57]. Consistent with this, we found that osthol induced greater nuclear expression of Nrf2 and antioxidant enzymes. Osthol also prevented the oxidation of lipids and proteins, as well as upregulation in the expression of Nox-4.

Nrf2 transcription factor not only reduces oxidative stress but also has additional metabolic effects, including detoxification, carbohydrate metabolism, fatty acid oxidation, and lipid metabolism [58]. Thus, Nrf2 upregulates lipases related to the degradation of triglycerides and phospholipids. It also negatively regulates enzymes involved in lipid biosynthesis and desaturation of fatty acids [58]. The present study shows that the preservation of renal lipid metabolism by the effect of osthol might also be mediated through its impact on Nrf2.

A limitation of this study is that osthol was given concurrently with the HF/HS diet from the beginning of the study. One also must be careful in extrapolating animal studies to the human situation. Nevertheless, the observation that osthol dose-dependently prevented renal injury and improved metabolic parameters suggests that it should be evaluated in clinical trials in humans.

## 4. Materials and Methods

### 4.1. Ethical Approval

This study was approved by the Internal Animal Care and Use Committee and conducted in adhesion with the current *Guide of Care and Use of Laboratory Animals* published by the National Institutes of Health (National Academies Press, 2011) and the Mexican Federal Regulation for animal experimentation and care (NOM-062-ZOO-2001) and for the disposal of biological residues (NOM-087-ECOL-1995). The study was also approved (16 December 2019) by the Institutional Animal Care and Use Committee of the Instituto Nacional de Cardiología Ignacio Chávez (INC/CICUAL/013/2019).

### 4.2. Experimental Protocol

We studied 4 male Wistar rats groups, weighing 220–240 g, that were kept in individual acrylic cages with paper nesting and bedding material (GreenSoft, RGS, Mexico City, Mexico) and a wood chip and a black acrylic holder as enrichment materials and were randomly assigned to one of the following groups (n = 6/group): (1) control (C), healthy animals receiving a normal diet and tap water; (2) HF/HS diet [31]^,^ and a sweetened beverage 11% (3.8% glucose, 7.2% fructose) (HF/HS); (3) HF/HS diet and 30 mg/kg of osthol (HF/HS + OT30); and (4) HF/HS diet and 40 mg/kg of osthol (HF/HS + OT40). The content of the HF/HS diet is specified in Table 1, and the fructose percentage used in the sweetened beverage has been previously reported and is similar to some soft-drink brands [32,33]. All groups were followed for 30 days, and at the end of follow-up, fasting animals were anesthetized with a low-flow vaporizer of isoflurane (Somno Suite, Kent Scientific Corpo-ration, Torrington, CT, USA) and euthanized by abdominal aorta exsanguination. Kidneys were collected and washed in cold phosphate-buffer saline (PBS) (pH 7.4) and frozen at −80 °C for further analysis.

### 4.3. Body Weight and Feeding

The rats’ body weight, fluid, and food intake were quantified and registered daily. Food was offered as a paste, containing 1 mL of water per gram of powdered diet, and the given amount was controlled and changed every week (week 1: 18 g; week 2: 20 g; week 3: 22 g; week 4: 24 g) in all groups. Osthol was dosed in the food accordingly to each rat’s daily body weight.

### 4.4. Systolic Blood Pressure (SBP)

The SBP was measured in conscious rats with a tail-cuff sphygmomanometer (IN125/R, ADI Instruments, Colorado Springs, CO, USA). The average of 3 measurements was reported.

### 4.5. Kidney Function

The glomerular filtration rate (GFR) was assessed with a transdermic monitor method using FITC-sinistrin injected via the tail. In brief, rats were anesthetized with 3% isoflurane, and the back was carefully shaved to place the transdermic monitor. FITC-sinistrin was injected via the tail vein at a dose 0.15 mg/g of body weight. To calculate FITC-sinistrin disappearance, data were collected during 2 h (as per the manufacturer’s recommendations). To convert the half-life excretion of FITC-sinistrin into the GFR, we used the following formula:

GFR (mL/min)/100g body weight = (21.33 mL/100g BW)/(t1/2(FITC-sinistrin) × (min)

Units were expressed as mg/mL/100 g of body weight. This method has been previously described [34,35,36]. Urine excretion of N-acetyl-β-d-glucosaminidase (NAG) was measured with a colorimetric method using 4-nitrphenyl-N-acetyl-β-d-glucosaminide as a substrate, where one unit of enzimatic activity represents the amount of enzyme that hydrolyses 1 µmol of substrate per minute at 37 °C, using a Synergy HT multi-mode microplate reader (BioTek Instruments, Inc., Winooski, VT, USA). Data were corrected by urinary volume [37]. Protein urine excretion was quantified by the Bradford method [38] and corrected by the 16 h urinary volume.

### 4.6. Fructokinase Activity

Fructokinase activity was indirectly assessed in the renal cortex by ATP consumption in the presence of fructose. In brief, kidney samples were homogenized in a buffer containing 20 mM Tris-HCl, 150 mM KCl, 1 mM EDTA, and 1 mM DTT (pH 7.5) using a polytron homogenizer and centrifuged for 10 min at 13,000 rpm at 4 °C. Renal homogenates were incubated in a buffer containing imidazole (50 mM)/KAc (1 M). Fifty mg of each lysate was exposed to 5 mM fructose and 1 mM ATP at 37 °C. ATP levels were measured after 2 h using a Synergy HT multi-mode microplate reader (BioTek Instruments, Inc., Winooski, VT, USA). The method has been previously reported [39].

### 4.7. Plasma Parameters

Quantification of glucose (Glucose-LQ, Spinreact, Girona, Spain), triglycerides (Triglycerides-LQ, Spinreact, Girona, Spain), and uric acid (Sekisui Diagnostics, Burlington, MA, USA) was performed using commercial kits according to the manufacturer’s instructions.

### 4.8. Intrarenal Fructose, Uric Acid, and Triglycerides

Renal cortex fructose was extracted from 100 mg of renal cortex with perchloric acid and measured with the anthrone method [40]. In brief, a reaction mix was preparated (sample or standard, sulfuric acid, and anthrone) and incubated at 37 °C for 50 min. The developed color was measured at 620 nm. Uric acid and triglycerides were extracted using 100 mg of renal cortex each, homogenized in a buffer containing 25 mM HEPES, 100 mM KCl, 1 mM DTT, 1 mM EDTA, and 5% NP-40 buffer for triglycerides with repetitive episodes of cold–heat shocks [41], and measured with the commercial kits used to quantify such parameters in plasma using a Synergy HT multi-mode microplate reader (BioTek Instruments, Inc., Winooski, VT, USA). The three parameters were corrected by milligrams of protein.

### 4.9. Western Blot Analysis

For the determination of the expression of different proteins, 60 mg of renal cortex was homogenized in MAP kinase lysis buffer [42], and 30 µg of protein was separated in 10% SDS-PAGE (Mini Protean II, Bio-Rad, Hercules, CA, USA) and transferred to a nitrocellulose membrane (Criterion Blotter, Bio-Rad, Hercules, CA, USA). After 1 h of blocking with non-fat dry milk (Bio-Rad, Hercules, CA, USA) in 5% TBS-Tween buffer. The antibodies used were as follows: fructokinase (Genetex, GTX109591, 1:5000 dilution), kidney injury molecule (KIM-1) (Genetex, GTX85067, 1:3000 dilution), fatty acid synthase (Genetex, GTX 13550, 1:2500 dilution), xanthine oxidase (Santacruz Biotechnology, sc398548 1:1000 dilution), Nox-4 (Novus Biologicals, NB-110-58851, 1:1000 dilution), nephrin (Genetex, GTX 31654, 1:10,000 dilution), IL-1β (Santacruz Biotechnology, sc-1250, 1:1000 dilution), IL-6 (Santacruz Biotecnology, sc-57315, 1:3000 dilution), phospho-AMPK Thr 172 (Cell Signaling, 40H9, 1:2000 dilution), Sirt-1 (Cell Signaling, D739, 1:2500 dilution), SREBP1 (Genetex, 79299, 1:3000 dilution), Nrf2 (Genetex, 103322, 1:1500 dilution), superoxide dismutase (Santacruz Biotechnology, sc101523, 1:1000 dilution), catalase (Santacruz Biotechnology, sc271358, 1:1500 dilution), glutathione peroxidase (Santacruz Biotechnology, sc22145, 1:1000 dilution), β-actin (Genetex, 109639, 1:10,000 dilution), proliferation cell nuclear antigen (PCNA; GTX100539, 1:2000 dilution), and neutrophil gelatinase associated-lipocalin (NGAL, Santacruz Biotechnology, sc-515876, 1:10,000 dilution). Chemiluminescense was visualized using horseradish peroxidase (HRP) secondary antibody (Cell Signaling, 7074) and ECL Clarity (Bio-Rad, Hercules, CA, USA). To analyze immunoblots, Image Studio Lite 5.2, Licor Biosciences software, was used.

### 4.10. Oxidative Stress Markers

Protein carbonylation and lipid peroxidation were evaluated using 100 mg of renal cortex by the colorimetric methods previously described [43]

Protein carbonylation: Renal cortex samples were homogenized in 20 mM PBS, incubated with 2,4-dinitrophenylhydrazine (DNPH), washed with ethanol-ethyl acetate (1:1), and resuspended in guanidine-HCL The presence of carbonyl groups in the proteins was measured using the reaction with DNPH. The protein carbonyl groups were estimated by using the molar absorption coefficient of 22,000 Molar^−1^cm^−1^ for DNPH derivatives, and their concentrations were expressed as nmol carbonyl groups/mg protein. Guanidine solution was used as a blank.

Lipid peroxidation: 4-Hydroxynonenal (4-HNE) was measured using a standard curve of tetramethoxypropane. A solution of 1-methyl-2-phenylindole in acetonitrile:methanol (3:1) was added to renal cortex homogenates, and the reaction was started with 37% HCl or methanesulfonic acid plus FeCl_3_ to measure 4-HNE. The optical density was measured at 586 nm after 1 h of incubation at 45 °C using a Synergy HT multi-mode microplate reader (BioTek Instruments, Inc., Winooski, VT, USA). Data were expressed as nmol of 4-HNE per milligram of protein (nmol HNE/mg protein).

### 4.11. Reagents

All the reagents used were from Millipore-Sigma (St. Louis, MO, USA), until otherwise established.

### 4.12. Statistical Analysis

Data were analyzed using GraphPad Prism 7 software (San Diego, CA, USA). Results are presented as the mean ± SD and were analyzed by one-way ANOVA. Statistical significance was established as (a) *p* < 0.05 vs. C, (b) *p* < 0.05 vsHF/HS, (c) *p* < 0.05 vs. OT30, and (d) *p* < 0.05 vs. OT40. Post hoc analysis was performed using Bonferroni;s test.

## 5. Conclusions

Osthol ameliorated metabolic syndrome manifestations induced by intake of a Western diet. Osthol also blocked kidney dysfunction, oxidative stress, and renal lipid accumulation, and this may be relateed to blocking KHK activity and stimulating Nrf2 production. As *Angelica* extracts have demonstrated to provide nephroprotection in acute and chronic kidney disease patients [59,60,61], we recommend clinical studies to determine whether the nutraceutical osthol might be an ancillary therapy to slow the progression of metabolic syndrome and kidney damage induced by an HF/HS diet.

## Figures and Tables

**Table 1:** Composition of regular and HF/HS diets.

Diet	Composition (% from Total Weight)	Nutritional Value (% of Total Kilocalories)
Regular	Commercially available: Lab Diet’s rodent diet 5010	Protein 28.66 Fat 13.11 Carbohydrate 58.22
HF/HS	1% Cholesterol 0.5% Cholic acid 5% Butter 30% Powdered sugar 10% Casein 2% NaCl 51.5% Regular diet	Protein 23.10 Fat 20.93 Carbohydrate 56.46

## Data Availability

The data presented in this study are openly available in FigShare at https://figshare.com/s/edd7db60220c719fb8b1 (accessed on 30 December 2020).

## Supplementary Materials

The following are available online at https://www.mdpi.com/1422-0067/22/5/2431/s1.
