# Supplementary material for: Osthol Ameliorates Kidney Damage and Metabolic Syndrome Induced by a High-Fat/High-Sugar Diet
Source: Int J Mol Sci. 2021 Feb 28;22(5):2431. doi: 10.3390/ijms22052431 (PMC7957708; doi:10.3390/ijms22052431)
Supplement: Supplementary file 1 [file ijms-22-02431-s001.pdf]

Supplementary materials

Skeletal muscle assessment:

Tissue was obtained from the left hind leg and homogenized in RIPA buffer with a polytron. Twenty µg of protein were loaded in PAGE-SDS gel, transferred in a nitrocellulose membrane, blocked with Non-fat dry milk 5% and incubated with primary antibody GLUT4 (Genetex, GTX33221, 1:5000 dilution) IRS1 (Genetex, GTX78916, 1:5000 dilution), IRS2 (Genetex, GTX57160, 1:5000 dilution) and beta-actin (Santacruz Biotechnology, sc-8432, 1:1000 dilution) overnight. ECL Plus Western Blotting was used to transfer chemiluminescence.

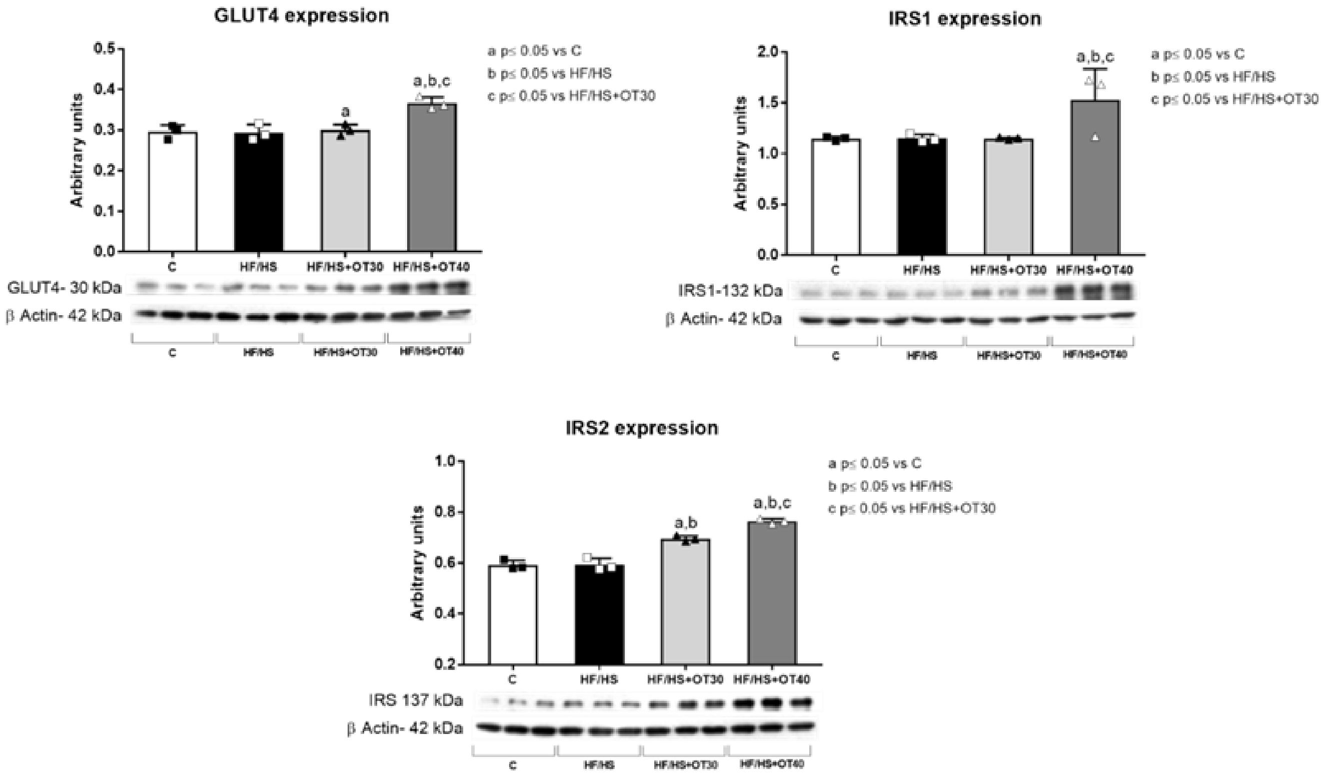

Figure S1: Expression of GLUT4, insulin receptor substrate (IRS) 1 (IRS1), and IRS2 in soleus muscle. For western blotting, three randomly selected samples per group were analyzed. Results are presented as mean ± SD and analyzed by one-way ANOVA. Statistical significance was established as a= $p<0.05$ vs C, b= $p<0.05$ vs AD, c= $p<0.05$ vs OT30. Post hoc analysis was performed using the Bonferroni test.

Liver assessment:

Tissue was obtained from the right lobe and homogenized in RIPA buffer with a polytron. Twenty µg of protein were loaded in PAGE-SDS gel, transferred in a nitrocellulose membrane, blocked with Non-fat dry milk 5%, and incubated with primary antibody KHK (Genetex, GTX109591, 1:10,000 dilution) and beta-actin (Santacruz Biotechnology, sc-8432, 1:1000 dilution) overnight. ECL Plus Western Blotting was used to transfer chemiluminescence.

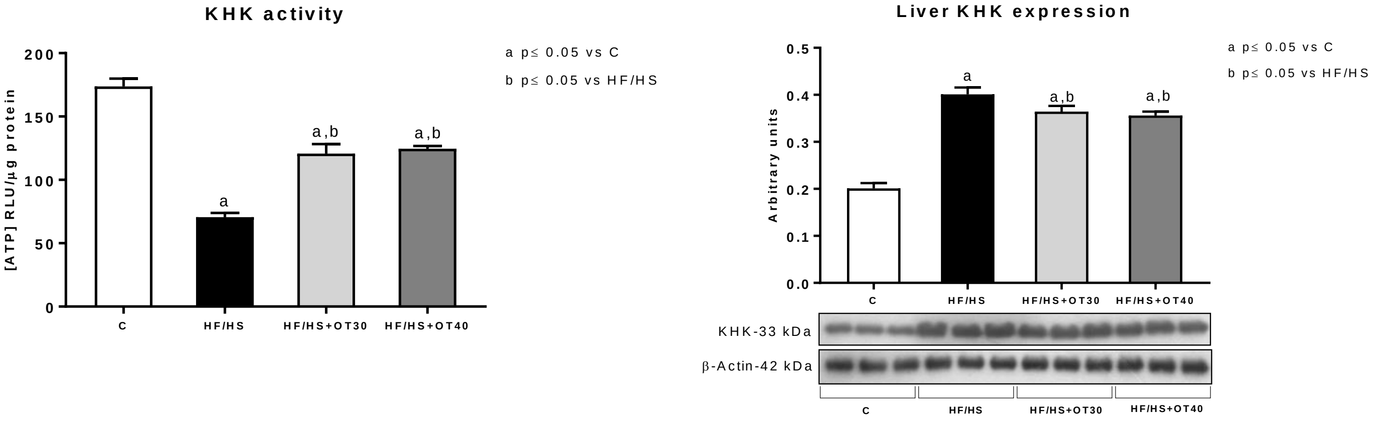

Figure. S2. Fructokinase assessment in liver tissue. Hepatic KHK activity and KHK expression. For western blotting, three randomly selected samples per group were analyzed. Results are presented as mean ± SD and analyzed by one-way ANOVA. Statistical significance was established as **a**= p<0.05 vs C, **b**= p<0.05 vs AD. Post hoc analysis was performed using the Bonferroni test.
